# Combined PARP Inhibition and Immune Checkpoint Therapy in Solid Tumors

**DOI:** 10.3390/cancers12061502

**Published:** 2020-06-09

**Authors:** Florent Peyraud, Antoine Italiano

**Affiliations:** 1 Department of Medical Oncology, Institut Bergonié, 33000 Bordeaux, France; peyraud.florent@gmail.com; 2 University of Bordeaux, 33076 Bordeaux, France; 3 Early Phase Trials and Sarcoma Unit, Institut Bergonié, 33000 Bordeaux, France

**Keywords:** PARP inhibitor, DNA damage response, immunotherapy, immune checkpoint inhibitor, PD-1, PD-L1, CTLA-4, combination therapy, solid tumors

## Abstract

Genomic instability is a hallmark of cancer related to DNA damage response (DDR) deficiencies, offering vulnerabilities for targeted treatment. Poly (ADP-ribose) polymerase (PARP) inhibitors (PARPi) interfere with the efficient repair of DNA damage, particularly in tumors with existing defects in DNA repair, and induce synthetic lethality. PARPi are active across a range of tumor types harboring *BRCA* mutations and also *BRCA*-negative cancers, such as ovarian, breast or prostate cancers with homologous recombination deficiencies (HRD). Depending on immune contexture, immune checkpoint inhibitors (ICIs), such as anti-PD1/PD-L1 and anti-CTLA-4, elicit potent antitumor effects and have been approved in various cancers types. Although major breakthroughs have been performed with either PARPi or ICIs alone in multiple cancers, primary or acquired resistance often leads to tumor escape. PARPi-mediated unrepaired DNA damages modulate the tumor immune microenvironment by a range of molecular and cellular mechanisms, such as increasing genomic instability, immune pathway activation, and PD-L1 expression on cancer cells, which might promote responsiveness to ICIs. In this context, PARPi and ICIs represent a rational combination. In this review, we summarize the basic and translational biology supporting the combined strategy. We also detail preclinical results and early data of ongoing clinical trials indicating the synergistic effect of PARPi and ICIs. Moreover, we discuss the limitations and the future direction of the combination.

## 1. Introduction

Over the past decade, poly(ADP-ribose) polymerase (PARP) inhibitors (PARPi) and monoclonal antibodies that block immune checkpoints, such as programmed cell-death 1 (PD-1) and cytotoxic T lymphocyte antigen 4 (CTL-4), have transformed the treatment of multiple types of cancers. Immune checkpoint inhibitors (ICIs) used as stand-alone therapeutic interventions give rise to durable objective responses in patients affected by a variety of cancers and have been approved for an ever-growing list of malignancies, including melanoma, non-small cell lung carcinoma (NSCLC), renal cell carcinoma, head and neck squamous cell carcinoma (HNSCC), and Hodgkin’s lymphoma [1,2,3,4,5,6,7]. More recently, monotherapy with PARPi as a maintenance strategy showed significant clinical activity in several cancer types harboring germline loss-of-function BRCA mutations such as ovarian, breast and pancreatic cancer [8,9,10,11]. However, despite these substantial advancements in clinical care, the majority of patients receiving either PARPi or ICIs alone do not provide benefit and a rationale to combine these treatments has emerged [12,13].

The groundbreaking success of anticancer immunotherapy is primarily based on the features of cancer cells and their ability to potentially initiate an antitumor immune response. These notable features include gene mutations resulting in abnormal protein expression patterns, such as neoantigens or tumor-associated antigens (TAAs) [14]. TAAs represent self-antigens that are aberrantly expressed or overexpressed in tumor cells, whereas neoantigens refer to non-self-antigens arising as a result of somatic mutation [14,15]. The formation of mutation-derived TAAs and neoantigen, reflecting the mutational burden of the tumor, allow the immune system to recognize tumor cell and initiate the cancer-immunity cycle [16,17]. The subsequent antitumor immune process against neoantigens relies on several steps, including the release and presentation of cancer antigens by antigen-presenting cells (APC), priming and activation of T cells, trafficking and infiltration of T cells, and recognizing and killing cancer cells [18]. However, a subset of cancer cells can escape host immune destruction by impairing one or more steps and result in tumor progression [19]. The role of immunotherapy is to reinvigorate antitumor immune response by disrupting co-inhibitory T cell signaling, transferring additional tumor-specific T cells clones and reshaping the immunosuppressive microenvironment [20,21,22,23]. Several strategies, including ICIs, adoptive T cell transfer, and vaccination, have been put to use in multiple cancers [24,25,26,27]. Nevertheless, due to complex and constantly evolving interactions between cancer cells and the immune system, both primary and acquired resistance with ICIs monotherapy are observed [28,29]. Therefore, combination treatment with ICIs is an attractive strategy to potentiate efficacy and lower resistance.

Recent molecular profiling of DNA damage repair genes has allowed the implementation of novel therapeutic strategies. By interfering with efficient DNA damage repair, the inhibition of PARP that target the base excision repair (BER) pathway leads to insufficient DNA repair, with subsequent unsustainable DNA damage, and thus represents a synthetic lethal therapeutic approach for the treatment of cancers with compromised ability to repair double-strand DNA breaks by homologous recombination (HR), including those with defects in *BRCA1/2* [30]. The unrepaired-DNA promotes immune priming through a range of molecular mechanisms and also leads to adaptative upregulation of programmed death ligand 1 (PD-L1) expression [31]. Moreover, PARPi modulates the inflammatory immune microenvironment of tumors and reinstates a productive T_H_1 immune response [31]. This multifaceted immunological effect of PARPi might be favorable to boost an antitumor immune response and enhance the efficacy of ICIs. In this review, we summarize the basic and translational biology supporting the combined strategy and provide a focus on preclinical studies and ongoing clinical trials of ICIs combined with PARPi, as well as perspectives and potential challenges of this combination strategy.

## 2. DNA Damage and PARP Inhibition

### 2.1. Role of PARP in DNA Damage Response

Cells are continuously faced with endogenous and exogenous stress that can ultimately lead to DNA damage. To preserve genomic integrity and prevent emergence of cancer, detection and repair of DNA is a critical process, managed by multiple pathways [32]. DNA single-strand break (SSB) damage is fixed by three main pathways: (1) BER, (2) nucleotide excision repair (NER), and (3) mismatch-repair (MMR). Possibly more dangerous DNA double-strand breaks (DSB) are restored by two additional pathways: (1) HR and (2) non-homologous end joining (NHEJ) [33]. Anomalies observed in DNA damage response (DDR) key genes, such as BRCA1/2 or TP53, are associated with cancer-prone phenotypes [34]. As a consequence, failure in DDR in an accurate and well-timed manner can result in the defective elimination of genome mutations and increases the risk of oncogenesis after established DNA damage events [35]. Depending on the context, cancer cells often harbor a lessened repertoire of DDR signaling competences, rendering them more reliant on a subset of DNA repair pathways and therefore more susceptible to DDR inhibition than normal cells [36].

PARP1/2 enzymes are core DNA-damage sensor and signal transducer in DDR, which bind to DNA breaks and catalyze the synthesis of poly(ADP-ribose)(PAR) chains on target proteins (PARylation) in the vicinity of the DNA break and itself (autoPARylation) [37]. These negatively charged PAR chains promote chromatin remodeling, recruit DNA repair-related protein complex and affect the replication fork progression speed [38,39]. The binding of PARP1 via zinc finger domains to sites of DNA-damage carries a conformational change in the PARP1 proteins and relieves the autoinhibitory interaction between the catalytic domain and helical domain. Then, the PARP1 co-factor nicotinamide (β-NAD^+^) is used as a substrate at the active site of the enzyme to catalyze the transfer of ADP-ribose moieties onto target proteins. The synthesis of ADP-ribose polymeric chains on proteins in the vicinity of DNA breaks, called PARylation, likely mediates DNA repair by modifying chromatin structure and by localizing DNA repair effectors [40]. Thereafter DNA-damage restore, autoPARylation occur that rapidly dissociate PARP from damage site [41]. The role of PARP has been well identified in BER-mediated SSB repair pathways, as well as other DDR pathways [42].

### 2.2. The Lethal Synthetic Effect of PARP Inhibitors

#### 2.2.1. Mechanism of Action of PARPi

**Figure 1:**
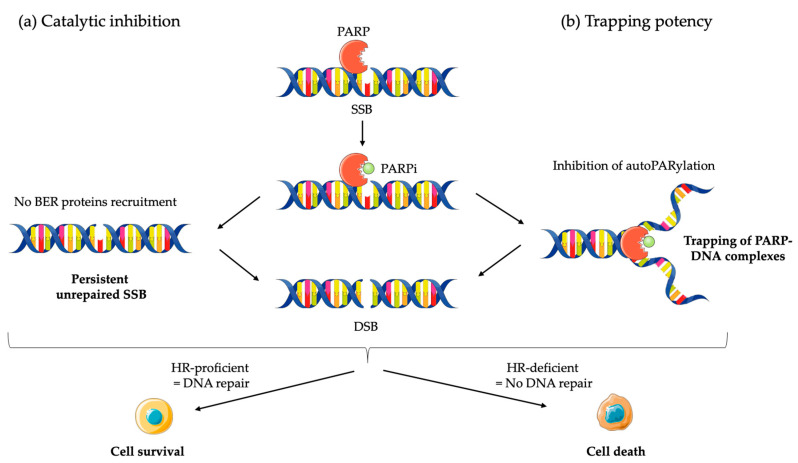
Mechanisms of action of PARP inhibitors (PARPi): (**a**) PARPi impedes PARP enzyme activity (or catalytic inhibition) and interferes with repair of single strand breaks (SSB) by disrupting the base excision repair (BER) pathway; (**b**) PARPi also causes trapping of PARP proteins on DNA by inhibiting autoPARylation. In homologous recombination (HR) proficient normal cells, DNA is repair and cell survive. The result in unresolved DNA double strand breaks (DSB) in HR deficient cells leads to cell death.

Although the precise mechanism by which PARPi kill tumor cells remains to be fully clarified, the anticancer effect is attributed to catalytic inhibition of PARP that block repair of DNA SSB [43] (Figure 1a). While PARPi is well-tolerated by normal cells, this effect of PARPi is more likely observed in tumor cells with a *BRCA*-deficient background [43]. As a result of defective enzymatic function induced by PARPi, the accumulation of SSB is subsequently encountered by replication forks and generates potentially lethal DSBs that need to be fixed [43,44]. In normal cells, the accumulation of DSBs are repaired preferentially by HR rather than NHEJ [45]. HR is a high-fidelity repair pathway that utilizes the sister copy of the damaged DNA as a template, leading to the reconstitution of the original sequence [46]. In contrast, NHEJ is intrinsically error-prone, modifies the broken DNA ends, and ligates them together with little or no homology, generating deletions or insertions [47]. However, in some cancer cells lacking BRCA1 or BRCA2, two key tumor suppressor proteins involved in DSB repair by HR, loss of PARP function leads to the accumulation of DSBs that are unrepaired or unsustainably repaired by NHEJ which results in cell death [44,48]. Based on the discovery of this synthetic lethality between *BRCA* and PARP, numerous PARPi have been developed, including olaparib, rucaparib, niraparib, talazoparib, and veliparib, which are mainly applied in cancer patients with *BRCA1/2* mutations [8,9,10,11,42]. 

Although the greatest efficacy of PARPi has been observed in tumors with *BRCA1/2* mutations, accumulating data indicate that synthetic lethality is inadequate to explain the whole antitumor activity. First, the ability of PARPi to inhibit PARP catalytic activity is poorly correlated to its cell-killing ability in HR deficiencies (HRD) cells [43,49]. In addition, PARPi induces cytotoxicity to a greater extent than PARP depletion [50,51]. Furthermore, PARP itself is essential to the cytotoxic effects of PARPi [52]. Actually, these facts may be attributed in part to the PARP trapping potency of PARPi (Figure 1b). Although the precise mechanisms of PARP trapping remains unclear, it has been proposed that PARPi could either prevent the release of PARP1 from DNA by inhibiting autoPARylation [53]. Likewise, PARPi binding to the catalytic site could cause allosteric changes in the PARP1 structure enhancing DNA avidity [49]. The trapping DNA-PARP complex stalls the progress of replication fork and elicit cytotoxic effects primarily through the conversion of unrepaired SSBs into lethal DSBs [43,49]. Moreover, PARPi could also act via the upregulation of NHEJ pathway, which presumably leads to genomic instability and eventual lethality [54]. Finally, PARPi could suppress the role of PARP in reactivating DNA replication forks and cause cell death [43]. Additional studies further demonstrated that loss of other tumor suppressor DNA repair proteins, many of which are involved in HR, such as RAD51, ataxia telengiectasia Rad3-related (ATR), ataxia telangiectasia mutated (ATM), checkpoint kinase 1 (CHK1), checkpoint kinase 2 (CHK2), and partner and localizer of *BRCA2* (PALB2) also caused sensitization to PARPi [49,55]. These results suggested that PARPi might be a useful therapeutic strategy not only for the treatment of *BRCA*-mutated tumors but also for the treatment of a wider range of non-*BRCA*-mutated tumors that are inherently HR deficient (HRD) or “BRCAness/HRDness” [56].

#### 2.2.2. Clinical Applications of PARPi 

The early development of PARPi focused initially on their use in combination with cytotoxic chemotherapy agents and radio-sensitizing drugs, but this was rapidly rejected because of excessive toxicity [57,58]. The potent antitumor effect of PARPi was originally observed in tumors harboring germline *BRCA1/2* mutations (*gBRCA1/2*m), such as familial breast and ovarian cancer [59]. This rapid translation of preclinical studies into promising clinical data triggered the development of several PARPi in different tumors types. Initially, PARPi in the clinic improved clinical benefits for germline or somatic *BRCA*-deficient ovarian cancer [60,61]. Subsequently, breast, pancreatic and prostate cancers that harbors defects in *BRCA* also demonstrated to be PARPi responsive [9,10,11,60,61,62]. More recently, it has been suggested that patients without *BRCA* mutations shared therapeutic vulnerabilities, especially tumors with deficiencies in HR. Indeed, the activity of PARPi is based on the concept of synthetic lethality, where an underlying HRD in tumor cells makes the cells highly susceptible to PARP inhibition [42]. This hypothesis has been further confirmed with multiple clinical studies showing that sensitivity to PARPi occurs in tumors beyond those with *BRCA* mutations, especially in HRD-positive tumors [63,64,65,66,67]. To date, five PARPi have been approved or orphan drug designed by the FDA (veliparib, rucaparib, talazoparib, niraparib, olaparib) and applied in clinical practice [42]. 

Despite the advances of PARPi in a particular population, acquired resistance is a common clinical phenotype. Owing to extensive preclinical studies, several resistance mechanisms have been identified that can be classified into four main categories. Firstly, numerous different mechanisms result in the reactivation of HR function. For example, secondary reversion mutations in several key HR repair (HRR) genes, such as *BRCA1/2*, *RAD51C/D,* and *PALB2*, restore the open reading frame and thus HR competency [68]. Moreover, the loss of p53-binding protein 1 (53BP1), a protein promoting NHEJ, is associated with PARPi resistance by recovery of HRR in *BRCA1*-deficient tumors [69]. By directly impacting the activity and abundance of PAR chains that decreased PARP trapping, mutations in DNA-binding domains of PARP1 and mechanisms that increase PARylation of PARP1 could also lead to PARPi resistance [70,71]. Furthermore, the cellular availability of the inhibitor is a critical step for successful therapy, as illustrated by the upregulation of ATP-binding cassette (ABC) transporters, such as the P-glycoprotein (PgP) efflux pump that have been described to reduce the efficacy of PARPi [72]. At last, restoration of replication fork protection that induces the stabilization of stalled forks may lead to PARPi resistance [73]. Indeed, fork degradation induced by PARPi is mediated by PTIP and EZH2 proteins, which upon loss lead to protection of the fork from nucleases and thereby resistance [74,75].

Intense preclinical and clinical research are ongoing in order to broadening responding patients, overcoming acquired resistance and enhancing the efficacy of PARPi [73]. The development of combination therapy encompassing PARPi is a potential approach to address these objectives. In addition to the hypothesis that patients with HRD tumors are more prone to produce neoantigen and exhibit higher mutational load, there is a preclinical rational suggesting that PARPi may promote the formation of neoantigen and generate tumor cell recognition by the immune system, making this class of drugs a potential partner for combination with ICIs [76,77].

## 3. The Revolution of Cancer Immunotherapy and Immune Checkpoint Inhibitors

Immunotherapy is proving to be an effective therapeutic approach in a variety of cancers [78]. In the last decade, the use of therapeutic antibodies that disrupt negative immune regulatory checkpoints and unleash pre-existing antitumor immune responses have achieved impressive clinical successes. Among the different types of cancer immunotherapy, ICIs have demonstrated the broadest impact by leveraging the cytotoxic potential of the human immune system, especially tumor-specific cytotoxic T cells. The role of T cells is critical to adaptative immunity and contribute to improved outcomes in a large range of cancers [79]. The activation of naïve T cells requires two distinct signals [80]. The generation of the first signal occurs by binding of major histocompatibility complex (MHC)-presented immunogenic peptide antigen to the heterodimeric T cell receptor (TCR). The transduction of the second signal, also referred as co-stimulation signal, arises through ligation of the T cell co-stimulatory surface receptor CD28 to its ligand CD80 (also known as B7-1) or CD86 (also known as B7-2) on the surface of professional antigen-presenting cells (APCs). Subsequent to both these signals, activated T cells begin to express co-inhibitory cell surface receptors that control T cell function, such as CTLA-4 and PD-1. The balance between co-stimulatory and co-inhibitory signals is crucial for the activation and tolerance of T cells [81]. Importantly, targeting these co-inhibitory pathways with ICIs in the context of cancer effectively shifts that balance toward activation, thereby overcoming tumor immune subversion [82].

### 3.1. CD80/86-CTLA-4 Signaling Pathway

The first negative regulator of T cell activation to be identified was CTLA-4, a co-inhibitory receptor that is constrictively expressed on Tregs and transiently upregulated during the course of T cell activation in peripheral lymphatic organs [83]. Bound by the same ligands (CD80/86) that provide co-stimulatory signals through CD28 but with higher affinity, CTLA-4 mainly impedes acquisition of T cell effector function by mediating transendocytosis and degradation of the ligands [84]. In addition, CTLA-4 delivers inhibitory signals that block T cell proliferation and secretion of IL-2, leading to T cell tolerance through induction of energy [85,86]. Moreover, CTLA-4 counterbalance TCR/CD3-mediated phosphorylation through immunoreceptor tyrosine-based inhibitory motif (ITIM) and impede the signal transduction of TCR [87]. Therefore, CTLA-4 engagement in numerous T lymphocyte populations operates as a cardinal immune checkpoint that ultimately hampers the acquisition of T cell effector functions and dampens the antitumor immune response.

### 3.2. PD-1/PD-L1 Signaling Pathway

The expression of PD-1 on activated immune cells is ubiquitous, including T cells, B cells, natural killer (NK) cells, and dendritic cells (DC), and yields inhibitory signals through binding of its two ligands, namely PD-L1 and PD-L2 [88]. Moreover, both PD-1 ligands are expressed on a wide variety of immune and non-immune cells [88]. More particularly, PD-L1 is found on a broad range of tissues and could be upregulated under inflammatory conditions such as cancers [89]. The expression of PD-L1 on the surface of tumors underlies the crucial relevance of the PD-1/PD-L1 pathway to neoplasm. Upon binding of TCR with antigen presented by MHC, PD-1 is engaged with its ligand and becomes functional. PD-1 activation leads to phosphorylation of the ITIM and immunoreceptor tyrosine-based switch motif (ITSM) in the PD-1 cytoplasmic tail and subsequently drive the recruitment of protein tyrosine phosphatase, such as Src homology region 2 domain containing phosphatase 1/2 (SHP1/2). As a consequence of dephosphorylation, these phosphatases antagonize positive signals that occur through the TCR nad CD28, affecting downstream signaling pathways. For example, TCR signaling molecules, such as Lck and ZAP-70, and co-stimulatory signaling cascades, such as PI3K-Akt-mTOR and Ras-MEK-ERK pathways, are inhibited. The impairment of these crucial signaling pathways alters the activation, proliferation, survival, cytokine production, metabolism, and epigenetic programs in T cells [90,91,92]. Tumors can exploit this pathway to escape T cell-mediated tumor-specific immunity.

### 3.3. Clinical Application of ICI

Since the recent success of antibodies targeting checkpoint molecules CTLA-4, PD-1, and PD-L1, the field of cancer immunotherapy has been experiencing a renaissance. The anti-CTL-4 inhibitor ipilimumab was the first ICI to obtain approval in 2011 for the treatment of metastatic melanoma [1,93]. Thereafter, ICIs have yielded broad clinical activity, leading to regulatory approval of several monoclonal antibodies in a variety of advanced and up-front disease settings, including melanoma, non-small cell lung, renal cell carcinoma, urothelial carcinoma, HNSCC, Merkel cell carcinoma, gastric carcinoma, hepatocellular carcinoma, Hodgkin’s lymphoma, as well as for any MMR-deficient/microsatellite instability (MSI) positive tumors [2,3,4,5,6,94,95,96,97,98,99,100,101,102,103,104,105]. However, as with PARPi, only a subset of patients derives benefit and a series of biomarkers have been developed to predict efficacy of ICI and select patients before treatment beginning.

Although the current understanding of the clinical response of ICI therapy indicates that there cannot be a single predictive biomarker, several factors have been identified as the core determinants of the efficacy of ICIs, such as tumor mutation burden (TMB) and particular mutational signature, the number of tumor-infiltrating lymphocytes (TILs), PD-L1 expression, immunosuppressive microenvironment, and MMR deficiency (MMRd) [106]. For example, tumors that harbor MMRd or some specific defects in DDR pathways beyond MMR demonstrated a higher ICI response [103,104,107]. The improve outcome in these patients is believed to be a result of increased mutational load, leading to greater immunogenicity. In addition, a novel perspective has arisen with the development of ICI-based combination therapy in order to improve ICI efficacy and overcome resistance. These include combinations with other checkpoint inhibitors, radiation therapy, chemotherapy, and targeted therapies, so as to foster antigen presentation, broadening T cell repertoire, impairing immunosuppressive elements, and increasing antitumor immune response [108,109].

## 4. Combination of PARPi and ICI Therapy 

### 4.1. A Rational to Combine PARPi and ICI

#### 4.1.1. Tumor Mutation Burden and Neoantigen

The mutational load in a tumor, termed as TMB and determined by the number of non-synonymous single nucleotide variants (nsSNVs), may impact the odds of generating immunogenic peptides and has been significantly correlated with ICI response in previous studies [110,111,112]. Even if the optimal TMB cut-off remains blurred across tumor types, the relationship with efficacy of ICI is robust [113,114]. TMB is considered as a surrogate of neoantigen load which predicts the therapeutic response of ICI [14,15,115]. Likewise, a growing amount of data indicate a closely association between TMB and DDR deficiency [116]. Highly mutated tumors often exhibit one or several mutations in key components of DDR or replicative pathways, including *MSH2* for MMR, *BRCA1/2* for HR and *POLE* for DNA replication, and correlate with ICI response [116]. In addition, patients with cancer harboring innate deficiencies in DDR genes, including MMR and HRR genes, achieved durable benefit from ICIs compared with patients without these deficiencies [116,117]. These results suggest that loss of normal DNA repair fidelity, such as DDR phenotype, may contribute to increased mutational load and neoantigens burden which affect the response to immunotherapy in these tumors. One relevant strategy in patients with HRD or others defects in DDR would be to combine PARPi and ICI.

**Figure 2:**
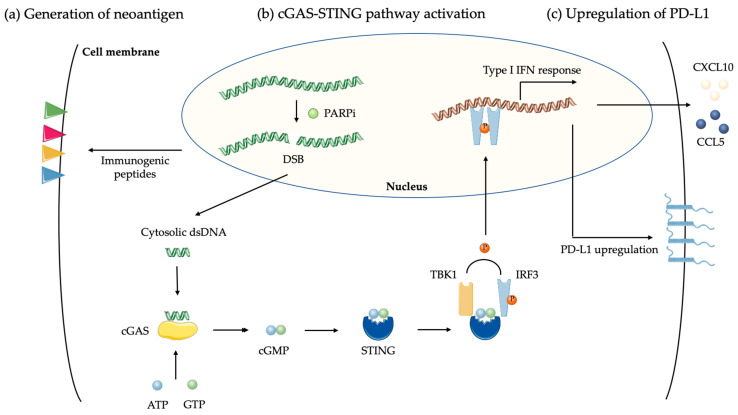
Biological effect of PARPi on cancer cells. (**a**) PARPi delays DNA repair that generates double-strand breaks (DSB) and catastrophic unrepaired DNA damages in tumor cells, increasing neoantigens load and tumor mutation burden; (**b**) DSB induced by PARPi in tumor cells produce double-strand DNA (dsDNA) fragments that, through cyclic GMP-AMP synthase (cGAS) binding, lead to stimulator or interferon genes (STING) activation and the generation of a type I interferon (IFN) response. This upregulates chemokines CCL5 and CXCL10 leading to T cell recruitment; (**c**) Upregulation of PD-L1 via STING pathway lead to T cell exhaustion.

Direct evidence that the targeting of DSB repair proteins with DDR inhibitors provoked and increased TMB is only beginning to emerge [118]. By affecting the HR pathway in tumor cells, impaired DNA repair induced by PARPi could subsequently generate catastrophic DNA damage that would increase the neoantigen load and TMB, thus driving a response to ICI and theoretically broadening the responding population (Figure 2a). Although tumors with non-MMR DDR genes deficiency, such as *BRCA1/*2 and other HR-related genes, have increased TMB, the association is weaker than that observed in MMR deficiency. Thereby, other fundamental links in tumor immunogenicity may be involved to explain the higher response rate to ICI in these patients [119,120].

#### 4.1.2. DNA Damages and cGAS-STING Pathway 

Aside from TMB, an emerging body of evidence supports a role for non-neoantigen-based mechanisms of tumor cell recognition and targeting by the host immune system. Genomic instability in tumor cells leads to the accumulation of incompletely repaired DNA damage, generating tumor-derived double-strand DNA (dsDNA) in the cytoplasm [121]. The sensing of tumor-derived dsDNA by cytosolic DNA sensor cyclic GMP-AMP synthase (cGAS) plays a major role in the activation of the stimulator of interferon (IFN) genes (STING) signaling pathway [122]. After the recognition of tumor-derived DNA within the cytosol, cGAS activates STING via the generation of 2′-5′ cyclic GMP-AMP (cGAMP). In turn, STING prompts phosphorylation and nuclear translocation of type I IFN transcriptional regulatory factors TANK-binding kinase 1 (TBK1) and IFN regulatory factor 3 (IRF3) [123,124]. Moreover, STING activates NF-*κ*B pathway which cooperates with IRF3. As a result, the upregulation of type I IFN promotes systemic immune response and regulates multiples components in anticancer immunity, especially T cells, NK cells and DCs [125]. According to recent studies, DNA damages and DDR deficiencies induce the activation of STING and NF-*κ*B pathways, leading to inflammation and infiltration of tumors by immune cells across multiple types of cancers, a prerequesite of tumor-killing effect of ICI [126,127,128,129,130].

In clinical practice, the antitumor activity of PARPi has been observed in patients with platinum-sensitive tumors regardless of BRCA1/2 mutation or HRD status, suggesting an alternative mechanism unrelated to conventional lethal synthetic-mediated cytotoxic effects [60]. The use of PARPi treatment leads to unresolved DNA lesions and to the production of cytosolic dsDNA fragments. The accumulation of cytosolic DNA activates in turn the DNA sensing cGAS-STING pathway and boosts production of type I interferon to induce antitumor immunity independently of DNA repair deficiency [131,132,133] (Figure 2b). These critical changes amplify STING signaling and its associated-transcription programs, thereby promoting TILs and antitumor immunity [132,133]. Moreover, it leads to increased levels of chemokines, such as CXCL10 and CCL5, that induce the activation and function of cytotoxic CD8^+^ T cell [132,133]. In addition, these effects of PARPi are further enhanced by ICI, providing a mechanistic rationale for the use of PARPi as immunomodulatory agents to harness the therapeutic efficacy of immunotherapy [134].

#### 4.1.3. PD-L1 Upregulation by PARPi 

A key mechanism underlying cancer immune evasion is the expression of inhibitory ligands, notably PD-L1, on the surface of cancer cells. Despite the approval of PD-L1 expression on tumor cells as a companion diagnostic for anti-PD1 therapy for patients with NSCLC, it remains an imperfect predictor of ICI response [4,135,136]. Via STING pathway, tumor-associated inflammation mainly drives the upregulation of immunosuppressive PD-L1 expression, thus reflecting the status of tumor immune microenvironment [137] (Figure 2c). In addition, defects in BRCA1/2 correlates to higher levels of PD-L1 expression [126,138]. Furthermore, the serine/threonine protein kinase glycogen synthase kinase 3β (GSK3β), a regulator of glycogen metabolism, interacts with PD-L1 and modulates its expression by inducing proteasome degradation of PD-L1 [139]. Based on the latter observation, preclinical models have unveiled that PARPi upregulates PD-L1 expression primarily through GSK3β inactivation in a dose-dependent manner, suppressing T-cell activation and increasing tumor cell killing [76]. Further explorations have shown that targeting DDR proteins PARP with PARPi significantly increased expression of PD-L1 [133]. Another report has demonstrated that PARPi-induced DSBs upregulate PD-L1 by ATM-ATR-CHK1 pathway independently of the IFN pathway [140]. Interestingly, subsequent combination therapy with ICI induced PARPi sensitization and led to a greater antitumor activity than either drug alone, putting forward a rational for combining PARPi with ICI as a useful therapeutic strategy [76].

#### 4.1.4. Reprogramming of Immune Microenvironnement by PARPi

In addition to altering the intrinsic immunogenicity of tumor cells through modulation of surface phenotype and intracellular pathways, DNA damage and deficient DDR pathways also modify the extrinsic immunogenicity of tumors at the level of microenvironment. As aforementioned, tumors with existing defects in DNA repair promote inflammation and T_H_1 immune response through a range of molecular mechanisms, leading to extrinsic tumor suppression [19]. However, despite the ability of DNA damage to contribute to tumor immune elimination, sustaining low-level DNA damage continues to foster inflammatory signaling that stimulates the infiltration by suppressive immune cells, like myeloid-derived suppressor cells (MDSCs) or tumor-associated macrophages (TAMs), which leads to further DNA damage via free radical release. This transformation boosts chronic inflammation, immunosuppression, and cancer progression [141,142]. PARPi may have the potential to shift from chronic, low level, DNA damage to more significant T_H_1 immune response and create a more susceptible tumor microenvironment [143]. Nevertheless, the self-sustaining cycle of DNA damage and chronic inflammation, which is challenging to overcome with PARPi single therapeutic approach, could potentially be addressed through combination with ICIs. 

In the wake of these biological findings, deficiencies in the HRR pathway and/or the use of DDR agents such as PARPi appear to activate immunosuppressive pathways, thus offering targetable immunological vulnerabilities in tumors. The interaction between DDR and immune response provides the basis of the combination therapy of ICI and PARPi. Thereby, combining ICIs and PARPi, which target HRD or induce a state of “BRCAness” in HR-proficient tumors, is a thrilling strategy, particulary as these agents have distinct and mostly non-overlapping toxicities [144,145]. Based on these assumptions, combining PARP inhibition with agents that have complementary mechanisms, such as ICIs, is currently subject to clinical testing.

### 4.2. Preclinical Data and Clinical Studies

#### 4.2.1. Combination of PARPi with Anti-PD1/PD-L1 ICIs

The first preclinical study evaluating PARPi veliparib in combination with anti-PD1/PD-L1 in the BRCA1-deficient BR5 mouse ovarian cancer model observed no significant boost in T cell activity and no improvement in survival [146]. However, the disappointing lack of activity for the combination with anti-PD1/PD-L1 in this preliminary work contrasts with more recent studies. In other preclinical studies conducted on breast cancer cell lines and xenograft models, PARPi olaparib significantly upregulated PD-L1 expression independently of cGAS-STING-IFN pathway and decreased antitumor immunity by attenuating the cell-killing activity of activated human peripheral blood mononuclear cells [76]. Further investigation in EMT6 syngeneic murine models demonstrated more potent antitumor effect and higher TILs infiltration with combined PARPi olaparib with anti-PD-L1 compared to either therapy alone [76]. In another mice model bearing BRCA1-deficient ovarian tumors, anti-PD1 monotherapy exhibited a non-significant effect and PARPi monotherapy delayed tumor progression compared to control group, whereas combination therapy significantly slowed the tumor growth and prolonged survival time [147]. A recent report indicated that coadministration of PARPi niraparib and anti-PD-1 enhanced the infiltration of immune cells into tumor microenvironment and increased synergistic antitumor activities in both immunocompetent BRCA-proficient and BRCA-deficient models, including breast cancer, lung squamous cell carcinoma, colon adenocarcinoma, bladder cancer, and sarcoma [148]. Similarly, additionally to augment CD8^+^ T cell infiltration, the association of PARPi olaparib and PD-L1 blockade induced complete tumor regression in multiple immunocompetent small cell lung carcinoma (SCLC) mice models [133]. These contrasting results may be explained by the use of different models, with disparities in immune contexture. While differences in anti-PD1/PD-L1 activities cannot be excluded, the use of PARPi with differential catalytic inhibition and various PARP trapping potencies may also explicate these discrepancies [52]. Taken together, the available translational and preclinical data clearly support the combination of PARPi and ICI. 

Based on these encouraging preclinical studies, several clinical trials have been conducted and some data are available to date (Table 1). In metastatic castration-resistant prostate cancer (mCRPC), the combination therapy of olaparib and durvalumab induced PSA responses (reduction ≥ 50%) in eight out of 17 patients (47%)(NCT02484404) [149]. Patients with DDR mutations exhibited greater benefit than those without known alterations (12-month progression-free survival probability of DDR-deficient vs. DDR-proficient, 83.3% vs. 36.4%, *p* = 0.031), suggesting DDR-deficient as a predictive biomarker of response [149,150]. In heavily pretreated platinum-resistant recurrent ovarian cancer, durvalumab and olaparib had clinical activity, irrespective of BRCA mutation status (NCT02484404) [151]. Interestingly, correlative analysis of paired pre- and on-therapy fresh core biopsy and blood samples collected on the latter cohort of recurrent ovarian cancer found that treatment enhanced IFN-γ and CXCL9/CXCL10 expression, systemic IFN-γ/TNF-α production and TILs, creating a more immunostimulatory milieu [152]. While tumoral and peripheral IFN-γ increases was correlated with durable clinical benefit from combined therapy, elevated circulating VEGFR3 levels were associated with worse progression-free survival (PFS), suggesting that VEGF/VEGFR pathway may act to counterbalance immunostimulatory changes induced by PARPi and would serve as a target to further improve efficacy of the combination [152]. Despite major limitations surrounding this exploratory analysis, no significant changes in TMB, PD-L1 or STING expression were noted in ovarian cancer patients treated with olaparib and durvalumab combination, thus warranting further investigation on the underlying biological mechanism [152]. On the other hand, the results of relapsed SCLC cohort of phase 2 NCT02484404 basket study durvalumab in combination with olaparib did not meet the preset bar for efficacy [153]. The preexisting TILs level, assessed by immunohistochemistry, seemed to predict tumor responses, suggesting a contribution from an immune-mediated response. The inflamed-phenotype at baseline, defined by high TILs infiltration, may help to identify patients who are most likely to respond to ICIs [153].

The phase 2 MEDIOLA basket trial assessed the efficacy and safety of chemo-free combination of olaparib and durvalumab in patients with solid tumors, including ovarian cancer, breast cancer and gastric cancer (NCT02734004). In gBRCAm platinum-sensitive relapsed ovarian cancer, the effect of the latter combination demonstrated an overall response rate (ORR) of 63% and a 12-week disease control rate (DCR) of 81% [154]. In gBRCAm HER2 negative metastatic breast cancer, the DCR was 80% at 12 weeks and 50% at 28 weeks, with ORR of 63% [155]. Median PFS (mPFS) was 9.2 months and median overall survival (mOS) was 21.5 months [155]. Moreover, patients with no prior line of chemotherapy had higher ORR and longer OS than those with two prior lines (respectively 78% vs. 50% for ORR and 21.3 vs. 16.9 months for OS) [155]. In platinum-resistant relapsed gastric cancer, the ORR was 10% with the 12-week DCR was 26% [156]. 

In the phase 2 TOPACIO trial (NCT02657889), niraparib and prembrolizumab combination therapy has demonstrated clinical benefit in platinum-resistant ovarian cancers and triple-negative breast cancers, with numerically higher response rates in those with BRCA-mutated tumors only in breast cancer cohort (ORR of BRCAm vs. BRCA wild-type in breast cancer cohort, 47% vs. 11%) [157,158]. However, the ovarian cohort of the TOPACIO study did not meet its primary endpoint of ORR.

The phase 1a/b PARPi pamiparib combined with anti-PD1 tislelizumab in patients with advanced solid tumors was associated with antitumour responses and clinical benefit (ORR of 20%)(NCT02660034) [159]. Similarly, the phase 1b/2 JAVELIN PARP Medley (NCT03330405) of avelumab plus talazoparib in advanced solid tumors is ongoing but showed preliminary antitumor activity and a manageable safety profile [160]. 

In the recent phase 2 NEODURVARIB trial (NCT03534492), durvalumab plus olaparib administered prior surgery of resectable muscle-invasive bladder cancer (MIBC) demonstrated a pathological complete response rate of 44.5%, suggesting an active and well-tolerated neoadjuvant strategy [161]. 

Except for the pamiparib–tislelizumab association, where an increased rate of immune-related hepatitis was observed, all combinations were well tolerated, with toxicities in line with those detected for the relevant drugs in monotherapy settings [159]. Numerous clinical trials are ongoing in a broad range of cancers that will help to decipher the exact role of PARPi with anti-PD1/PD-L1 combination strategy (Table 2). 

#### 4.2.2. Combination of PARPi with Anti-CTLA-4 ICIs

Contrary to the in-depth attention paid to anti-PD1/PD-L1, the association of PARPi with anti-CTLA-4 is less studied, probably due to the misunderstood biological effect of PARPi on CTLA-4 signaling pathway and T cell effector functions. However, a previous study has unveiled that increased tumor immunogenicity modulates the response to CTLA-4 blockade [110]. Furthermore, BRCA dysfunction is associated with increased T cell recruitment to tumour site and higher expression of immune response genes [162,163,164]. Moreover, targeting DDR proteins through PARP inhibition may stimulate antigen presentation and immunogenicity via increased TMB and T cell cytotoxic activity [118]. Hence, one might surmise that tumors harboring BRCA dysfunction and treated with PARPi could increase tumoral immunogenicity, thus sensitizing the tumor to anti-CTLA-4 antibodies. All together, these data provide a rationale for the combination of PARPi with anti-CTLA-4 in BRCA-deficient tumours.

Initial preclinical study conducted on an immunocompetent BRCA1-deficient BR5 murine ovarian cancer model revealed that anti-CTLA-4 combined with PARPi veliparib enhanced IFN-γ production and effector/memory T cell infiltration [146]. In addition, CTLA-4 antibody synergized therapeutically with the PARPi, resulting in long-term survival in a majority of mice [146]. To date, no other preclinical study employing this combination in solid tumors has been released, and based on these data, clinical studies were developed.

Preliminary results from a phase 1 study combining olaparib and tremelimumab for the treatment of women with BRCA-deficient recurrent ovarian cancer demonstrated evidence of therapeutic effect with acceptable tolerability (NCT02571725) [165]. Ongoing clinical trials will help to figure out the promising antitumor activity of PARPi with anti-CTLA-4 monoclonal antibodies (Table 2).

#### 4.2.3. Combination of ICI with Others DDR Inhibitors: Moving beyond PARP in Targeting the DDR

In light of the evidence that unrepaired DNA damage induced by PARPi expands the anti-tumor activity of the ICI, the therapeutic landscape of DDR-targeting agents has promptly unfolded to include inhibitors of other key mediators implied in DNA replication and repair, such as ATM, ATR, Chk1, Chk2, DNA-PK, and WEE1 [166]. The crucial roles of ATR and ATM protein kinases in DDR signaling involve the maintenance of replication fork stability and the regulation of cell cycle control checkpoints by operating together via downstream targets Chk1 and Chk2, respectively [167,168]. Additionally, the kinase activity of DNA-PK is required for NHEJ and a distinct nuclear kinase WEE1 controls mitotic entry as well as nucleotide pools in coordination with DNA damage response, making these kinase as potential targets for cancer therapy [168,169]. 

The role of DDR inhibitors as immunomodulatory agents that possibly potentiate ICIs has recently emerged. Recent preclinical evidence suggested that ATR or ATR inhibitors exhibit immunomodulatory functions and enhances antitumor efficacy to immune checkpoint therapy. The combination of selective ATR inhibitor, avelumab, and platinum-based chemotherapy resulted in antitumor effect in syngeneic tumor models, leading to overall survival benefit compared to any dual-combination group, and also provided protective antitumor immunity with immunological memory in cured mice [170]. Likewise, a recent study demonstrated that pharmacological ATM inhibition induced a type I IFN-mediated innate immune response in pancreatic cancer model that is further enhanced by radiation and led to increased sensitivity to anti-PD1 therapy [171]. Moreover, a preclinical model of immunocompetent SCLC in vivo observed that Chk1 inhibition, a protein kinase implicated in DSB repair, potentiated the antitumor effect of PD-L1 blockade and augmented cytotoxic T cell infiltration [133]. Moreover, a potent and selective DNA-PK inhibitor, that selectively blocks the NHEJ for repair of DSB, induced an immunomodulatory phenotype and elevated the expression of PD-L1 protein via cGAS-STING pathway activation in irradiated p53-mutant cancer cells [172]. Concordantly, combination of DNA-PK inhibitor, radiotherapy and avelumab in syngeneic mice with p53-mutant cancer cells demonstrated a superior benefit and offers a new approach to combination radio-immunotherapy of cancer [172]. All this evidence together provides a clear rationale to combine other DDR pathway inhibitor with immunotherapies. 

Outside of PARPi, other DDR inhibitors are currently clinically tested in combination with ICI in several tumor types. A phase 1 modular study of ceralasertib, a potent and selective ATR inhibitor in combination with durvalumab is being evaluated in patients with advanced or metastatic cancers, including NSCLC and HNSCC (NCT02264678) [173]. Preliminary results of this study indicated acceptable tolerance with signals of activity [173]. Similarly, in the phase 1b BISCAY study, patients with metastatic MIBC with any HRD detected are being treated with durvalumab and olaparib or the WEE1 inhibitor adavosertib (NCT02546661) [174]. These ongoing trials will provide new insights into a combinatorial approach.

### 4.3. Future Perspectives

Although preclinical and clinical studies revealed interesting tumor responses with PARPi and ICIs in different tumors types, these combinations did not markedly improved antitumor effect compared to the individual agents alone. These disappointing outcomes suggest a lack of synergistic interaction of PARPi and ICIs. Several points could eventually explain these discrepancies.

Foremost, current animal models probably do not recapitulate the whole genomic heterogeneity or tumor microenvironment of human cancer. Indeed, data in mouse models do not predict response to the combination of PARPi and ICIs, thus limiting the transfer to Human. Better in vitro and in vivo models are needed to translate preclinical findings into clinical results. For example, the use of humanized mouse models could be a relevant strategy.

Concerning the nature and the magnitude of combination versus monotherapy benefit, most studies to date have relied on early endpoints such as ORR or DCR. The latter endpoints would be informative in case of patients with limited expected response to PARPi, such as tumors with DDR-proficient. However, in tumors where a high response to PARPi is expected, such as BRCA-mutated and other HRD phenotypes, it would be more relevant to assess the combination benefit in terms of the duration of response or survival, thus necessitating prolonged monitoring in such studies.

One another limitation in the interpretation of available data are the format of clinical trials. Indeed, current clinical results are only provided by non-randomized trials, which only allow cross-trial study comparisons. This approach of comparison is not methodologically and statistically acceptable to distinguish the specific role of each drug in terms of efficacy. To overcome this problem in the clinic, treatment strategies using DDR inhibitors with ICIs should be optimized through the use of randomized controlled multi arms phase III trials designed to enable the interpretation of the effect of each agent alone or in combination. Moreover, additional effort is required to determine the dose and schedule dependency of DNA repair modulation on the immune system.

Based on the synthetic lethality effect, the use of PARPi have been approved preferentially in tumors that harbor deficiency in the HR pathway, such as BRCA-mutated tumors and in a subsets of BRCA-negative HRD-positive cancer [175]. The assumptions that BRCA dysfunction is associated with the recruitment of T cell to tumor sites, and that PARPi may increase the immunogenicity of tumor cells have paved the way for combined strategy of ICIs and PARPi in BRCA-associated or more largely to HRD cancers. However, the combinatory effect of PARPi and ICIs in tumors without HR deficiency remains unknown. While in vitro studies in non-HRD cancer cell lines provide the rationale for a combined strategy, in vivo preclinical evidence suggesting that PARPi might increase the efficacy of ICIs has been conducted preferentially in BRCA-deficient tumor models, thus limiting the translational relevance. The question of whether PARPi and ICI should be restricted to non-HRD tumors or should be used more broadly has to be elucidated through the understanding of underlying biological mechanisms. Further work is needed to uncover the target population who are most likely to benefit from the combination strategy.

It is of note that most tumor types where the combination strategy was evaluated already demonstrated significant benefit of PARPi monotherapy, but limited activity for ICIs. Thereby, it would be more pertinent to evaluate the association in a population which cancer treatment represent a critical unmet medical need. For example, it would be interesting to focus on a subgroup which does not derive benefit or is primary/secondary resistant to either PARPi or ICI alone. Furthermore, dosing and scheduling of drugs largely differed across studies. The optimal dose and schedule of each agent needs to be determined with empirical clinical method as well as correlative analysis, including sequential tumor biopsies and serial blood collection.

In the future, a critical next step is to understand and identify the optimal patient population that will benefit the most from this combination. Biomarkers will likely play an even greater role in identifying those patients most likely to respond to PARPi. Whereas tumors with BRCA1/2 mutation or HRD are more likely to benefit from PARPi and ICI, an unmet medical need remains in the HR-proficient populations, so it is important to evaluate whether PARPi can sensitize these tumors to ICI in clinical settings. Moreover, obtaining contemporaneous tumor tissues and matched blood samples, associated with the integration of precision medicine, are key steps to better understand the mechanisms of action and the resistance pathways, and to identify novel predictive biomarkers of response. These crucial strides will allow a deeper comprehensive landscape of the interface between DNA damage and tumor immunity.

At last, although the current focus is on a combination of PARPi with anti-PD1/PD-L1 or anti-CTLA-4, other targeted agents moving beyond PARP in targeting DDR pathways as well as other promising immune-directed therapies are under development, and should be considered in the near future. 

## 5. Conclusions

Genomic instability is a key hallmark of cancer that arises notably owing to DDR deficiencies. Major breakthroughs have been made with the successful targeting of DNA repair in clinical oncology [166]. Alike, immune-modulating therapies have also reshaped the landscape of cancer medicine [176]. However, treatment with either PARPi or ICIs alone often do not translate into benefit. While defects in DDR pathways might potentially be considered as predictive biomarkers of ICIs response, compelling evidence has provided a biological rationale, and demonstrated synergistic benefit, for combining ICIs with DDR inhibitors such as PARPi. To date, many preclinical and clinical researches focus on the identification of other tumors or molecular subtypes of cancers in which this combination will ultimately have a clinical impact, and thus turning more non-responders into responders with a strikingly boosted depth and duration of response.

While PARPi-induced a tumor HRD phenotype as well as immune modulation represent a rational approach for the association with ICIs, the combination did not markedly enhance the antitumor effect compared with individual agents in to-date clinical trials. To bring forward a critical change and improvements in patient outcomes, the development of accurate and predictive biomarker should become a priority. Moreover, unraveling the different mechanisms of resistance to PARPi and ICIs is required to pave the way for novel combination strategies. In addition, the appropriate dosing and scheduling of each agent should be determined in order to minimize adverse events while maximizing benefit and outcomes. Finally, elucidating the role of and interplay between DDR pathways, the tumor immune microenvironment and inhibitor agents, such as PARPi and ICIs, will be critical to the success and future development for this combination.

## Figures and Tables

**Table 1:** Recent clinical trials of combination of PARPi and ICIs.

Tymor Type	Study Identifier	Setting	ICI Agent	PARPi Agent	Design	Patients	Primary Endpoint	Outcome
Ovarian	NCT02571725	Phase I	Tremelimumab (10 mg/kg Q4W)	Olaparib (300 mg BID)	*gBRCAm* recurrent ovarian cancer	3	Safety and RP2D	No DLT or grade 3 AE ORR 100% with 3 PRs
	NCT02484404	Phase II	Durvalumab (1500 mg Q4W)	Olaparib (300 mg BID)	Platinum-resistant recurrent ovarian cancer	35	Clinical activity (ORR)	ORR 14% with 5 PRs (irrespective of *BRCA* status), DCR 71%, mPFS 3.9 months Acceptable toxicity
	NCT02657889 (TOPACIO/KEYNOTE-162)	Phase II	Pembrolizumab (200 mg Q3W)	Niraparib (200 mg QD)	Platinum-resistant recurrent ovarian cancer	60	Clinical activity (ORR)	ORR 18% with 3 CRs and 8 PRs (irrespective of *BRCA* and HRD status), DCR 65% mPFS 3.4 months Acceptable toxicity
	NCT02734004 (MEDIOLA)	Phase II	Durvalumab (1500 mg Q4W)	Olaparib (300 mg BID)	*gBRCAm* platinum-sensitive ovarian cancer	32	Clinical activity (DCR)	12-week DCR 81%, ORR 63% with 6 CRs and 14 PRs Acceptable toxicity
Breast	NCT02657889 (TOPACIO/KEYNOTE-162)	Phase II	Pembrolizumab (200 mg Q3W)	Niraparib (200 mg QD)	Advanced/Metastatic TNBC	55	Clinical activity (ORR)	ORR 21% with 5 CRs and 5 PRs (stronger activity in *BRCA*-mutated tumors), DCR 49% Acceptable toxicity
	NCT02734004 (MEDIOLA)	Phase II	Durvalumab (1500 mg Q4W)	Olaparib (300 mg BID)	*gBRCAm* HER2 negative mBC	30 for clinical activity and 34 for safety	Clinical activity (DCR) and safety	12-week DCR 80%, 28-week DCR 80%, ORR 63%, mPFS 8.2 months, mOS 20.5 months (especially in chemotherapy-free patients) Acceptable toxicity
Prostate	NCT02484404	Phase II	Durvalumab (1500 mg Q4W)	Olaparib (300 mg BID)	Previously treated mCRPC	17	Clinical activity (rPFS) and safety	rPFS 16.1 months with 12-month rPFS 51.5% (especially in men with DDR abnomalities) Acceptable toxicity
SCLC	NCT02484404	Phase II	Durvalumab (1500 mg Q4W)	Olaparib (300 mg BID)	Relapsed SCLC	19	Clinical activity (ORR)	ORR 10.5% with 1 PRs and 1 CRs, clinical benefit 21.1% (preexisting TIL predictive of response) Acceptable toxicity
Bladder	NCT03534492 (NEODURVARIB)	Phase II	Durvalumab (1500 mg Q4W)	Olaparib (300 mg BID)	Resectable muscle-invasive bladder cancer	28	Clinical activity and safety	Pathological CR 44.5% Acceptable toxicity (grade 3 or higher AEs 8.3%)
Gastric	NCT02734004 (MEDIOLA)	Phase II	Durvalumab (1500 mg Q4W)	Olaparib (300 mg BID)	Platinum-resistant relapsed gastric cancer	39 for clinical activity and 40 for safety	Clinical activity (DCR) and safety	12-week DCR 26%, ORR 10% with 2 CRs and 2 PRs Unacceptable toxicity (grade 3 or higher AEs 48%)
Solid tumors	NCT02660034	Phase Ia/b	Tislelizumab (2 mk/kg Q2W)	Pamiparib (20, 40 or 60 mg BID)	Previously treated advanced solid tumors	49 patients	Safety and RP2D	DLT 8% with 23 immune-related AEs RP2D tislelizumab 200 mg and pamiparib 40 mg ORR 20% with 2CRs and 8 PRs
Solid tumors	NCT03330405 (JAVELIN PARP Medley)	Phase Ib/II	Avelumab (800 mg Q2W)	Talazoparib (1 mg QD)	Previously treated advanced solid tumors	34 patients	Safety and clinical activity (ORR)	First-cycle DLT 25% ORR 8% with 1 PR, SD 50%

Abbreviations: AE: adverse events; BID: twice a day; CR: complete response; DCR: disease control rate; DLT: dose-limiting toxicity; *gBRCAm*: germline *BRCA*-mutated; ICI: immune checkpoint inhibitor; mCRPC: metastatic castration-resistant prostate cancer; mOS: median overall survival; mPFS: median progression-free survival; ORR: overall response rate; PARPi: poly(ADP)-ribose polymerase inhibitor; PR: partial response; Q2W: every 2 weeks; Q3W: every 3 weeks; Q4W: every 4 weeks; QD: once daily; rPFS: median radiographic progression-free survival; RR: response rate according to RECIST v1.1, SD: stable disease; SCLC: small-cell lung carcinoma.

**Table 2:** Ongoing clinical trials of combination of PARPi and ICIs (www.clinicaltrials.gov).

PARPi	ICI		Study Identifier	Phase	Tumor Type and Conditions	Status
	Type	Drug				
**Olaparib**	**Anti-CTLA-4**	Tremelimumab	NCT02571725	I/II	*gBRCAm* recurrent epithelial ovarian, fallopian tube, or primary peritoneal carcinoma	Recruiting
		Tremelimumab	NCT02485990	I/II	Recurrent or persistent epithelial ovarian, fallopian tube, or primary peritoneal carcinoma	Not recruiting
		Tremelimumab	NCT04034927	II	Platinum-sensitive advanced epithelial ovarian, fallopian tube, or primary peritoneal carcinoma	Recruiting
	**Anti-PD1**	Pembrolizumab	NCT04209686	II	Locally advanced or metastatic gastric carcinoma	Not yet recruiting
		Pembrolizumab	NCT04306367	II	Locally advanced or metastatic cholangiocarcinoma	Recruiting
		Pembrolizumab	NCT02861573 (KEYNOTE-365)	I	Metastatic castration-resistant prostate cancer	Recruiting
		Pembrolizumab	NCT03740165 (KEYLYNK-001	III	*BRCA*-non-mutated advanced epithelial ovarian, fallopian tube, or primary peritoneal carcinoma	Recruiting
		Pembrolizumab	NCT03976323 (KEYLINK-006)	III	Metastatic non-squamous cell lung cancer	Recruiting
		Pembrolizumab	NCT04123366 (KEYLYNK-007)	II	HRR-mutated or HRD positive advanced or metastatic solid tumors	Recruiting
		Pembrolizumab	NCT03976362 (KEYLYNK-008)	III	Metastatic squamous non-small cell lung cancer	Recruiting
		Pembrolizumab	NCT04191135 (KEYLYNK-009)	II/III	Locally advanced triple negative breast cancer	Recruiting
		Pembrolizumab	NCT03834519 (KEYLYNK-010)	III	Metastatic castration-resistant prostate cancer	Recruiting
	**Anti-PD-L1**	Atezolizumab	NCT02849496	II	Locally advanced unresectable and or metastatic HER negative breast cancer	Recruiting
		Durvalumab	NCT03594396	I/II	Resectable stage II/III triple negative breast cancer	Recruiting
		Durvalumab	NCT03534492 (NEODURVARIB)	II	Resectable urothelial carcinoma	Recruiting
		Durvalumab	NCT03459846 (BAYOU)	II	Advanced or metastatic platinum-ineligible urothelial carcinoma	Not recruiting
		Durvalumab	NCT03951415 (DOMEC)	II	Recurrent, refractory or metastatic endometrial cancer or carcinosarcoma of the endometrium	Recruiting
		Durvalumab	NCT03851614 (DAPPER)	II	Locally advanced or metastatic mismatch repair proficient colorectal cancer, pancreatic adenocarcinoma or leiomyosarcoma	Recruiting
		Durvalumab	NCT03167619 (DORA)	II	Locally advanced or metastatic platinum-treated advanced triple negative breast cancer	Recruiting
		Durvalumab	NCT03544125	I	Metastatic triple negative breast cancer	Not recruiting
		Durvalumab	NCT03801369	II	Metastatic triple negative breast cancer	Recruiting
		Durvalumab	NCT04053322 (DOLAF)	II	Locally advanced or metastatic ER positive HER2 negative breast cancer	Recruiting
		Durvalumab	NCT03810105	II	DDR-mutated castration sensitive biochemically recurrent non-metastatic prostate cancer	Recruiting
		Durvalumab	NCT02882308 (OPHELIA)	II	Resectable squamous cell carcinoma of the head and neck	Completed
		Durvalumab	NCT03991832	II	IDH-mutated solid tumors (glioma, cholangiocarcinoma, and solid tumors)	Not yet recruiting
		Durvalumab	NCT03772561 (MEDIPAC)	I	Advanced or metastatic solid tumors	Recruiting
		Durvalumab	NCT02484404	I/II	Advanced, recurrent or metastatic ovarian, triple negative breast, lung, prostate, colorectal carcinoma or solid tumors	Recruiting
		Durvalumab	NCT03579784	II	Unresectable or recurrent gastric carcinoma	Recruiting
		Durvalumab	NCT03737643 (DUO-O)	III	Newly diagnosed advanced ovarian, fallopian tube or primary peritoneal carcinoma or carcinosarcoma	Recruiting
		Durvalumab	NCT04269200 (DUO-E)	III	Newly diagnosed advanced or recurrent endometrial carcinoma	Not yet recruiting
		Durvalumab	NCT03801369	II	Metastatic triple negative breast cancer	Recruiting
		Durvalumab	NCT03775486	II	Metastatic non-squamous cell lung cancer	Recruiting
		Durvalumab	NCT02734004 (MEDIOLA)	I/II	Advanced or metastatic solid tumors	Recruiting
		Durvalumab	NCT03842228	I	DDR-mutated unresectable, advanced or metastatic solid tumors	Recruiting
	**Anti-PD-L1 + Anti-CTLA-4**	Durvalumab + Tremelimumab	NCT04169841 (GUIDE2REPAIR)	II	HRR-mutated advanced or metastatic solid tumors (breast, lung, head and neck, clear cell renal, endometrial, ovarian, urothelial and prostate cancer	Not yet recruiting
		Durvalumab + Tremelimumab	NCT03923270	I	Extensive small cell lung cancer	Recruiting
		Durvalumab + Tremelimumab	NCT02953457	I/II	DDR-mutated recurrent or refractory ovarian, fallopian tube or primary peritoneal carcinoma	Recruiting
**Niraparib**	**Anti-CTLA-4**	Ipilimumab	NCT03404960 (Parpvax)	I/II	Advanced pancreatic cancer	Recruiting
	**Anti-PD1**	Cetrelimab	NCT03431350 (QUEST)	I/II	Metastatic castration-resistant prostate cancer	Recruiting
		Dostarlimab	NCT04068753 (STAR)	II	Recurrent or progressive cervix cancer	Recruiting
		Dostarlimab	NCT04313504	II	Reccurent or metastatic head and nead squamous carcinoma	Not yet recruiting
		Dostarlimab	NCT03016338	II	Recurrent or advanced endometrial cancer	Recruiting
		Dostarlimab	NCT03602859 (FIRST)	III	Stage III or IV non-mucinous epithelial ovarian, fallopian tube or primary peritoneal cancer	Recruiting
		Dostarlimab	NCT03955471 (MOONSTONE)	II	Advanced platinum-resistant ovarian, fallopian tube or primary peritoneal carcinoma	Recruiting
		Dostarlimab	NCT03806049	III	Advanced or recurrent platinum-sensitive ovarian, fallopian tube or primary peritoneal carcinoma	Not yet recruiting
		Dostarlimab	NCT03307785	I	Advanced or metastatic solid tumors	Not recruiting
		Dostarlimab	NCT03651206 (ROCSAN)	II/III	Reccurent or progressive uterine or ovarian carcinosarcoma	Not yet recruiting
		Nivolumab	NCT03404960 (Parpvax)	I/II	Advanced pancreatic cancer	Recruiting
		Pembrolizumab	NCT02657889 (TOPACIO)	I/II	Advanced or metastatic triple negative breast or ovarian cancer	Not recruiting
		PD-1 inhibitor	NCT03308942	II	Locally advanced or metastatic non-small cell lung carcinoma	Not recruiting
	**Anti-PD-L1**	Atezolizumab	NCT03695380	I	Advanced ovarian, fallopian tube or primary peritoneal carcinoma	Recruiting
		Atezolizumab	NCT03598270 (ANITA)	III	Recurrent ovarian, fallopian tube or primary peritoneal carcinoma	Recruiting
		Atezolizumab	NCT04185831 (MEGALiT)	II	Advanced or metastatic solid tumors	Not yet recruiting
	**Anti-PD-1**	Nivolumab	NCT04187833	II	*BRCA-* or *BRCAness*-mutated resectable or metastatic melanoma	Recruiting
		Pembrolizumab	NCT04158336	I/II	Solid tumors	Recruiting
	**Anti-PD-L1**	Avelumab	NCT02912572	II	Recurrent or persistent endometrial cancer	Recruiting
		Avelumab	NCT03964532 (TALAVE)	I/II	Advanced breast cancer	Recruiting
		Avelumab	NCT03637491	II	Locally advanced or metastatic RAS-mutant solid tumors	Recruiting
		Avelumab	NCT04173507 (A LUNG-MAP)	II	*STK11*-mutated recurrent or metastatic non-squamous non-small cell lung cancer	Recruiting
		Avelumab	NCT03565991 (JAVELIN BRCA/ATM)	II	*BRCA* or *ATM*-mutated locally advanced or metastatic solid tumors	Recruiting
		Avelumab	NCT04068831	II	Locally advanced or metastatic clear-cell renal cell carcinoma	Recruiting
		Avelumab	NCT04052204	II	Locally advanced or metastatic head and neck squamous carcinoma or CRPC	Recruiting
		Avelumab	NCT03330405 (JAVELIN PARP MEDLEY)	II	Locally advanced or metastatic solid tumors	Recruiting
		Avelumab	NCT03642132 (JAVELIN Ovarian PARP 100)	III	Locally advanced or metastatic ovarian cancer (NSCLC, triple negative breast cancer, HR+ breast cancer, recurrent platinum-sensitive ovarian cancer, urothelial cancer, CRPC)	Not recruiting
**Veliparib**	**Anti-PD-1**	Nivolumab	NCT02944396	I	Advanced or metastatic NSCLC	Completed
		Nivolumab	NCT03061188	I	Advanced, recurrent, refractory or metastatic solid tumors	Not recruiting
**Rucaparib**	**Anti-PD-1**	Nivolumab	NCT03572478	I/II	Metastatic CRPC or recurrent endometrial cancer	Recruiting
		Nivolumab	NCT03639935	II	Advanced or metastatic cholangiocarcinoma	Recruiting
		Nivolumab	NCT02873962	II	Relapsed ovarian, fallopian tube or peritoneal cancer	Recruiting
		Nivolumab	NCT03338790 (CheckMate 9KD)	II	Metastatic castration-resistant prostate cancer	Recruiting
		Nivolumab	NCT03522246 (ATHENA)	III	Newly diagnosed advanced ovarian, fallopian tube or primary peritoneal carcinoma or carcinosarcoma	Recruiting
		Nivolumab	NCT03824704 (ARIES)	II	Platinum-treated advanced ovarian, fallopian tube or primary peritoneal carcinoma or carcinosarcoma	Not recruiting
		Nivolumab	NCT03958045	II	Platinum-sensitive small cell lung carcinoma	Recruiting
		Nivolumab	NCT03995017 (RiME)	I/II	Unresectable or metastatic gastric or esophageal adenocarcinoma	Recruiting
		Pembrolizumab	NCT03559049	I/II	Metastatic NSCLC	Recruiting
	**Anti-PD-L1**	Atezolizumab	NCT03101280	I	Advanced or metastatic platinum-sensitive ovarian or endometrial cancer or triple negative breast cancer	Not recruiting
		Atezolizumab	NCT04276376 (ARIANES)	II	DDR-deficient or platinum sensitive solid tumors	Recruiting
		Atezolizumab	NCT03694262 (EndoBARR)	II	Recurrent progressive endometrial carcinoma	Recruiting
**Pamiparib**	**Anti-PD-L1**	Tislelizumab	NCT02660034	I	Advanced or metastatic solid tumors	Recruiting
